# AS1411-functionalized albumin nanoparticles for nucleolin-targeted realgar delivery and therapy in triple-negative breast cancer

**DOI:** 10.3389/fphar.2026.1891302

**Published:** 2026-07-29

**Authors:** Liping Dong, Wenzhuo Ran, Yanqiong Li, Lixia Yang, Xi Zhou

**Affiliations:** 1 School of Nursing, Changsha Medical University, ChangSha, China; 2 Hunan Provincial Key Laboratory of the Research and Development of Novel Pharmaceutical Preparations, ChangSha, China; 3 Department of Laboratory Medicine, Traditional Chinese and Western Medicine Hospital of Wuhan, Tongji Medical College, Huazhong University of Science and Technology, Wuhan, China

**Keywords:** AS1411 aptamer, bovine serum albumin, breast cancer, GADD45G/mTOR, realgar, targeted delivery

## Abstract

Realgar (As_4_S_4_) has shown antitumor activity, but its pharmaceutical application is limited by poor aqueous solubility, low bioavailability, and systemic toxicity. In this study, we developed AS1411-functionalized albumin nanoparticles (As_4_S_4_@ BSA-AS1411 NPs) for targeted delivery of realgar to triple-negative breast cancer (TNBC). Bulk As_4_S_4_ was converted into nanosized clusters and encapsulated in a bovine serum albumin (BSA) matrix, followed by surface conjugation with the nucleolin-targeting aptamer AS1411. The resulting nanoparticles showed suitable physicochemical properties and good colloidal stability in physiologically relevant media, together with enhanced cellular internalization in 4T1 cells. Compared with non-targeted nanoparticles, AS1411 modification significantly increased cytotoxicity and reduced the migratory ability of 4T1 cells. Mechanistic studies showed that treatment with As_4_S_4_@BSA-AS1411 NPs was associated with upregulation of GADD45G and downregulation of mTOR, suggesting a stress-related antiproliferative effect. *In vivo* fluorescence imaging demonstrated increased tumor accumulation of AS1411-functionalized NPs. In 4T1 tumor-bearing mice, systemic administration of As_4_S_4_@BSA-AS1411 NPs produced greater tumor growth inhibition than non-targeted controls, accompanied by increased apoptosis and reduced proliferative activity in tumor tissues. In addition, the formulation showed good *in vivo* tolerability, with no evident systemic toxicity or hemolytic activity. These findings indicate that AS1411-functionalized albumin NPs are a promising delivery system for improving the antitumor efficacy and safety profile of realgar in TNBC.

## 1 Introduction

Realgar (As_4_S_4_), an arsenical compound utilized in traditional Chinese medicine for over three millennia, demonstrates therapeutic effects beyond its historical applications for dermatological and parasitic conditions (Wu et al., 2011). Contemporary research has revealed its potent antitumor properties, which operate through pleiotropic mechanisms governing tumor growth and progression (Wang et al., 2008; Mao et al., 2010). However, the clinical application of realgar faces significant pharmacological challenges. Its poor aqueous solubility, limited bioavailability, and formulation instability substantially restrict its therapeutic utility (Li et al., 2023). Furthermore, long-term administration at elevated doses presents considerable toxicity risks, thereby limiting its clinical adoption (Zhu et al., 2018).

Advanced drug delivery systems offer promising solutions to these limitations. Such systems enable precise targeting and controlled release of therapeutic agents (Nie et al., 2020; Sun and Jiang, 2023). Previous investigations have successfully incorporated arsenic-based compounds into nanoliposomal and albumin-based carriers, demonstrating enhanced efficacy alongside reduced toxicity profiles (Chen et al., 2006; Zhang et al., 2022). The development of As_4_S_4_ nanoparticles (NPs) presents particular technical challenges. Conventional physical methods yield heterogeneous particle size distributions, while chemical approaches may introduce potentially toxic byproducts (Bujňáková et al., 2015). Consequently, bioinspired strategies that emulate endogenous transport systems (Dong et al., 2023), such as albumin-mediated delivery, have emerged as particularly promising alternatives.

Bovine serum albumin (BSA) represents an optimal carrier platform due to its biocompatibility, biodegradability, and demonstrated safety profile (Elzoghby et al., 2012). Nevertheless, passive accumulation alone is insufficient for effective tumor selectivity, necessitating additional molecular guidance systems (Elzoghby et al., 2012; Huang et al., 2023; Wang et al., 2024). Nucleic acid aptamers provide such targeting capability through their capacity to form specific three-dimensional conformations (Zhou and Rossi, 2017). The AS1411 aptamer, which specifically recognizes nucleolin overexpressed on malignant cells, facilitates efficient intracellular delivery through receptor-mediated endocytosis (Reyes-Reyes et al., 2010).

This investigation focuses on triple-negative breast cancer (TNBC), an aggressive malignancy characterized by absence of estrogen receptor, progesterone receptor, and HER2 expression. This molecular profile renders conventional targeted therapies ineffective, while chemotherapy frequently yields transient responses followed by rapid disease progression, underscoring the urgent need for alternative targeting strategies (Boyle, 2012; Bianchini et al., 2016; Zagami and Carey, 2022).

**SCHEME 1:**
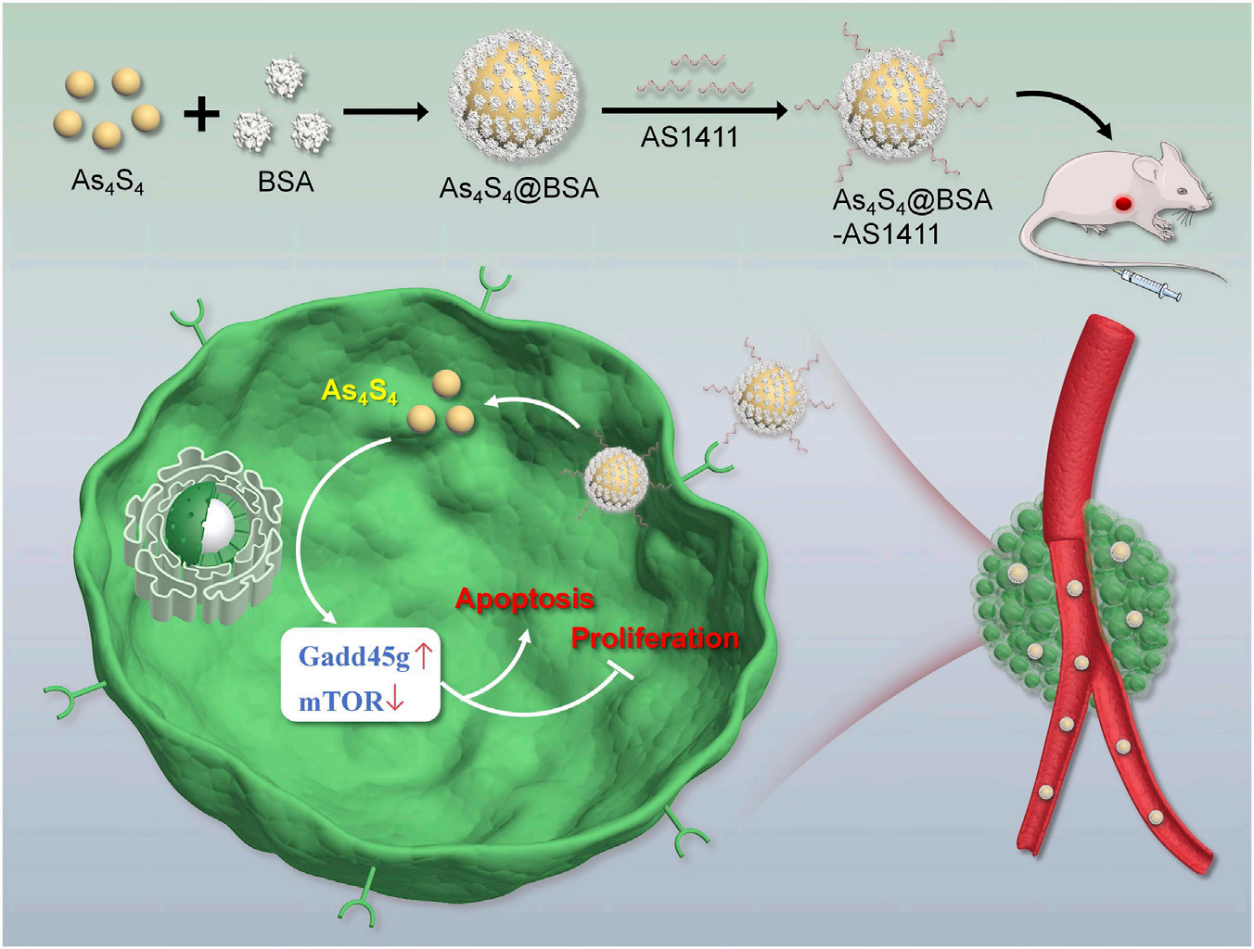
Schematic Illustration of the Preparation of As_4_S_4_@BSA-AS1411 NPs and Targeted Delivery for Cancer Therapy.

In this work, we report a targeted nanotherapeutic strategy that re-engineers As_4_S_4_, a classical mineral medicine with established antitumor activity yet long-standing pharmacological limitations, into a precision nanomedicine (Scheme 1). Focusing on TNBC, a highly aggressive malignancy lacking effective molecular targets, bulk realgar was transformed into nanoscale As_4_S_4_ clusters and encapsulated within a biocompatible bovine serum albumin matrix, thereby markedly improving its aqueous dispersibility and pharmacological controllability. Importantly, surface functionalization with the nucleolin-targeting AS1411 aptamer enables active tumor targeting, a feature particularly relevant for TNBC, where nucleolin is frequently overexpressed.

Unlike conventional arsenic-based formulations, this albumin–aptamer hybrid nanoplatform simultaneously addresses the key challenges of realgar therapy, including poor bioavailability, nonspecific toxicity, and insufficient tumor selectivity. We demonstrate that AS1411-mediated targeting markedly amplifies the antitumor efficacy of As_4_S_4_ while effectively mitigating systemic toxicity. Mechanistically, the targeted nanoparticles induce robust apoptosis and suppress tumor proliferation through modulation of stress-responsive and growth-regulatory pathways, notably involving GADD45G activation and mTOR inhibition.

Collectively, this study establishes a conceptual framework for the modernization of traditional mineral medicines through nanotechnology and molecular targeting, and highlights the potential of aptamer-guided, albumin-based nanocarriers to unlock the therapeutic value of pharmacologically challenging inorganic agents for safe and effective cancer treatment.

## 2 Materials and methods

### 2.1 Materials

BSA and tetramethylethylenediamine (TEMED) were purchased from Aladdin (Shanghai, China). Live/Dead Viability/Cytotoxicity Kit was obtained from Invitrogen (NY, USA). 4′,6-Diamidino-2-phenylindole (DAPI) was purchased from Solarbio (Beijing, China). 1-Ethyl-3-(3-dimethylaminopropyl) carbodiimide (EDC) and N-hydroxysuccinimide (NHS) were purchased from Sinopharm (Shanghai, China). Penicillin–streptomycin solution, RPMI-1640 medium, and MTT were obtained from Gibco (Gaithersburg, MD, USA). BCA protein assay kit and PBST (0.05% Tween 20 in 10 mM PBS) as well as phenylmethylsulfonyl fluoride (PMSF) were purchased from Dingguo Changsheng (Beijing, China). GADD45G antibody and ECL chemiluminescence detection kit were obtained from Hunan Auragene (Hunan, China). Alanine aminotransferase, aspartate aminotransferase, creatinine, and urea assay kits were purchased from Nanjing Jiancheng (Jiangsu, China). Acrylamide/bisacrylamide (30%, 29:1) solution was purchased from Biosharp (Anhui, China). DMEM and trypsin–EDTA were purchased from Gibco (New York, USA). All oligonucleotides were purchased from Shanghai Bioegene (Shanghai, China). The sequences were as follows:

AS1411: 5′-GGT​GGT​GGT​GGT​TGT​GGT​GGT​GGT​GG-3′; rAS1411 (scrambled control): 5′-TCT​ACT​ATT​GTG​TTG​TGT​ATA​ACT​TT-3′.

Scrambled aptamer (rAS1411)-modified nanoparticles were used as controls for uptake and biodistribution studies, whereas non-targeted As_4_S_4_@BSA NPs served as controls for efficacy and safety evaluations.

### 2.2 Preparation of As_4_S_4_@BSA-AS1411 NPs

Crude realgar was dissolved in ethylenediamine to prepare an As_4_S_4_ cluster solution (20 mg/mL) and centrifuged (15,000 rpm, 5 min) to remove insoluble precipitates. The supernatant (1 mL) was rapidly added to 8 mL ultrapure water, followed by dropwise addition of BSA (3 mL, 20 mg/mL) under stirring for 5 min. The pH was adjusted to ∼7.0 with 4 M HCl (7 mL) and stirred for 30 min. EDC (20 μL, 1 mg/mL), NHS (10 μL, 1 mg/mL), and AS1411 (10 μL, 10 μM) were then added sequentially and stirred for 60 min to obtain As_4_S_4_@BSA-AS1411 NPs. The nanoparticles were collected by centrifugation (20,000 rpm, 10 min), washed twice with ultrapure water, redispersed in ultrapure water, and stored at 4 °C.

### 2.3 Characterization of As_4_S_4_@BSA-AS1411 NPs

The morphology of the NPs was characterized by transmission electron microscopy (TEM). The particle size distribution and zeta potential were measured by dynamic light scattering (DLS). The formation of the As_4_S_4_@BSA-AS1411 colloidal system was confirmed by observing the Tyndall effect using a laser beam.

### 2.4 Encapsulation efficiency

For encapsulation efficiency (EE) measurement, As_4_S_4_@BSA-AS1411 NPs suspension (1 mL, 1 mg/mL) was digested with 10% nitric acid (6 mL) overnight at 70 °C. The digest was diluted to 10 mL with ultrapure water and filtered (0.45 μm PVDF). An arsenic standard was treated identically. Arsenic content was determined by atomic absorption spectroscopy (AAS) using an arsenic calibration curve, and EE was calculated as:

$$\text{EE }\left(\%\right)=\left(W_{1}/W_{2}\right)\times 100\%$$

where W_1_ is the mass of As_4_S_4_ encapsulated in nanoparticles and W_2_ is the total As_4_S_4_ initially added.

### 2.5 Stability evaluation

To evaluate colloidal stability, nanoparticles were incubated in ultrapure water, PBS (pH 7.4), or DMEM with 10% FBS at 37 °C, and size changes were monitored over time by DLS.

To evaluate the serum stability of AS1411 conjugated to the nanoparticles, As_4_S_4_@BSA-AS1411 NPs containing an equivalent amount of AS1411 and free AS1411 were incubated in 50% FBS at 37 °C for 0, 6, 12, 24, 48, and 72 h. At each time point, the nanoparticle suspensions were centrifuged to pellet the nanoparticles, and the supernatants were collected to detect any AS1411 released from the nanoparticles. The collected supernatants, together with the corresponding free AS1411 samples, were analyzed by 2% NA-Red agarose gel electrophoresis using 1 × TAE as the running buffer. Gel images were acquired using a gel imaging scanner.

### 2.6 Cellular uptake

For cellular uptake (live-cell imaging), AS1411 or rAS1411 was FAM-labeled. 4T1 cells (2 ×10^5^ cells/dish) were seeded in 35-mm glass-bottom dishes and cultured for 24 h. Cells were then incubated with As_4_S_4_@BSA-FAM-AS1411 NPs or As_4_S_4_@BSA-FAM-rAS1411 NPs (As_4_S_4_ = 5 μg/mL) in DMEM supplemented with 10% FBS for 4 h at 37 °C. To verify AS1411-mediated cellular uptake, a competitive inhibition assay was performed. Briefly, 4T1 cells were pre-incubated with excess free AS1411 aptamer (50 μM) for 30 min at 37 °C before nanoparticle treatment. The cells were then incubated with As_4_S_4_@BSA-FAM-AS1411 NPs at an equivalent As_4_S_4_ concentration of 5 μg/mL for another 4 h at 37 °C in complete DMEM. After washing twice with PBS (10 min each), nuclei were stained with Hoechst 33,342 (1 μg/mL) for 15 min, and cells were imaged live using a confocal fluorescence microscope (LSM780 NLO, Zeiss, Germany).

### 2.7 *In vitro* antitumor activity

4T1 cells (5,000 cells/well) were seeded in 96-well plates for 24 h and then treated with As_4_S_4_@BSA NPs or As_4_S_4_@BSA-AS1411 NPs at As_4_S_4_ concentrations of 0–40 μg/mL (0, 0.5, 1, 2, 5, 10, 20, 40 μg/mL; 0 μg/mL, PBS control; n = 5) for 24 h. Medium was replaced with 100 μL MTT solution (0.5 mg/mL) and incubated for 4 h. Formazan was dissolved in DMSO (150 μL/well) for 15 min with shaking, and absorbance was read at 490 nm. Cell viability was normalized to the PBS control.

4T1 cells were seeded in 35-mm glass-bottom dishes (2 × 10^5^ cells/dish) and cultured for 24 h. Cells were then treated with PBS, As_4_S_4_@BSA NPs, or As_4_S_4_@BSA-AS1411 NPs (As_4_S_4_ = 5 μg/mL) for 24 h. After treatment, cells were washed twice with PBS and stained with Calcein-AM and propidium iodide (PI) according to the manufacturer’s instructions. Briefly, cells were incubated with the staining solution for 15 min at 37 °C in the dark, washed with PBS, and imaged immediately using a fluorescence microscope. Live and dead cells were identified by green (Calcein-AM) and red (PI) fluorescence, respectively.

### 2.8 Transwell migration

Transwell migration was evaluated using 24-well inserts (8 μm pores). To exclude the possibility that reduced cell migration was caused by treatment-related cytotoxicity, cell viability was assessed in parallel under the same drug concentration and exposure duration used in the migration assay. Briefly, 4T1 cells were seeded in 96-well plates at a density of 5 × 10^3^ cells/well and cultured for 24 h. The cells were then treated with PBS, As_4_S_4_@BSA NPs, or As_4_S_4_@BSA-AS1411 NPs at an equivalent As_4_S_4_ concentration of 2 μg/mL for 12 h. Cell viability was measured using the MTT assay as described above and normalized to the PBS-treated control. For the migration assay, 4T1 cells were resuspended in serum-free medium and seeded into the upper chamber at 5 × 10^4^ cells/insert with PBS, As_4_S_4_@BSA NPs, or As_4_S_4_@BSA-AS1411 NPs (As_4_S_4_ = 2 μg/mL). The lower chamber contained 600 μL medium with 20% FBS as a chemoattractant. After 12 h at 37 °C, non-migrated cells were gently removed from the upper surface with a cotton swab. Migrated cells on the lower surface were fixed with 4% paraformaldehyde (15 min), stained with 0.2% crystal violet (20 min), washed with PBS three times (5 min each), and imaged. Migrated cells were quantified by counting cells in five randomly selected microscopic fields per insert. Each condition was tested in duplicate, and the assay was independently repeated three times.

### 2.9 Western blot analysis for GADD45G and mTOR protein

4T1 cells (1 × 10^6^ cells/well, 6-well plates) were treated with As_4_S_4_@BSA NPs or As_4_S_4_@BSA-AS1411 NPs (As_4_S_4_ = 5 μg/mL, 1 mL/well, serum-containing medium) for 24 h (PBS control; n = 3). Cells were lysed in RIPA + PMSF on ice (30 min) and centrifuged (12,000 g, 15 min, 4 °C). Equal protein (20 μg) was subjected to SDS-PAGE and transferred to PVDF membranes, followed by blocking (5% milk, 1 h) and incubation with HUABIO primary antibodies against GADD45G (1:1,000), mTOR (1:5,000), and β-actin (1:2,000) (overnight, 4 °C). Membranes were then incubated with HUABIO HRP-conjugated secondary antibodies (1:50,000), developed by ECL, and quantified by ImageJ with normalization to β-actin.

### 2.10 *In vivo* tissue distribution

All animal procedures were approved by the Changsha Medical University Animal Care and Use Committee (Approval No. X2025014). We evaluated the *in vivo* biodistribution and tumor accumulation of As_4_S_4_ nanoparticles functionalized with Cy5.5-labeled AS1411 (or rAS1411) in a 4T1 tumor-bearing nude mouse model. Female BALB/c nude mice (6 weeks) were inoculated subcutaneously in the right axilla with 4T1 cells (1 × 10^6^/100 μL PBS). Tumor volume was calculated as V = (L × W^2^)/2. When tumors reached ∼300 mm^3^, mice (n = 3 mice/group) received tail-vein injections of As_4_S_4_@BSA–Cy5.5-AS1411 NPs, As_4_S_4_@BSA–Cy5.5-rAS1411 NPs, or PBS ([As_4_S_4_] = 10 mg/kg; same Cy5.5 dose). Mice were anesthetized with pentobarbital and imaged at 1 and 24 h (KODAK; Ex/Em = 690/790 nm), then euthanized by cervical dislocation under deep anesthesia for *ex vivo* imaging of tumors and major organs.

### 2.11 *In vivo* pharmacodynamic study

Tumor growth was monitored after inoculation. When the mean tumor volume reached ∼100 mm^3^, nude mice were randomly assigned to three groups (n = 5): PBS, As_4_S_4_@BSA NPs, and As_4_S_4_@BSA-AS1411 NPs. Formulations were administered by tail-vein injection (100 µL) at an As_4_S_4_ dose of 10 mg/kg at 3-day intervals (days 0, 3, 6, 9, and 12; five doses).Tumor length (L) and width (W) were measured every other day, and tumor volume was calculated as V = (L × W^2^)/2. Three days after the final dose, mice were euthanized by cervical dislocation under deep anesthesia; tumors were excised, photographed, and weighed.

Apoptosis in tumor tissues was assessed by TUNEL staining on formalin-fixed, paraffin-embedded sections. After deparaffinization and rehydration, sections were permeabilized with proteinase K (20 μg/mL, 37 °C, 20 min) and stained using a commercial fluorometric TUNEL kit according to the manufacturer’s instructions, in which TdT incorporates red fluorescent dUTP at DNA strand breaks. Sections were incubated at 37 °C for 1 h in a humidified chamber, counterstained with DAPI, mounted with antifade medium, and imaged by fluorescence microscopy. TUNEL-positive nuclei showed red fluorescence, and all nuclei were stained blue with DAPI.

Proliferation was evaluated by Ki67 immunofluorescence on formalin-fixed, paraffin-embedded tumor sections. After deparaffinization and rehydration, antigen retrieval was performed in citrate buffer (pH 6.0) by microwave heating. Sections were blocked with 5% normal goat serum for 30 min and incubated overnight at 4 °C with anti-Ki67 antibody (1:200). After washing, sections were incubated with an Alexa Fluor 594-conjugated secondary antibody for 1 h at room temperature in the dark, counterstained with DAPI, mounted with antifade medium, and imaged by fluorescence microscopy. Ki67-positive nuclei showed red fluorescence.

### 2.12 Safety assessment

*In vivo* safety was evaluated by monitoring body weight, serum biochemistry, and histology. Body weight was recorded every other day during the study. On day 15 after the first administration, blood was collected via orbital sampling. A portion of the whole blood was collected into EDTA-containing anticoagulant tubes for routine hematological analysis, while the remaining blood was processed to obtain serum for biochemical analysis (ALT, AST, Cre, and BUN). Mice were then anesthetized with pentobarbital and euthanized by cervical dislocation under deep anesthesia. Major organs (heart, liver, spleen, lungs, and kidneys) were harvested, fixed in 4% paraformaldehyde, sectioned, and stained with H&E for histopathological examination.

### 2.13 Hemolysis assay

A hemolysis assay was performed to assess hemocompatibility. Heparinized mouse blood was centrifuged (3,000 rpm, 10 min, 4 °C), and RBCs were washed three times with PBS (pH 7.4) and resuspended to 4% (v/v). NPs were prepared in PBS at As_4_S_4_ concentrations of 0, 10, 20, 50, 100, and 200 μg/mL. RBC suspension (250 μL) was mixed with nanoparticle solution (250 μL) and incubated at 37 °C for 3 h with gentle shaking. After centrifugation, supernatants (100 μL) were analyzed at 570 nm. PBS and 0.1% Triton X-100 served as negative and positive controls, respectively. Hemolysis (%) = [(A_sample_ -A_negative_)/(A_positive_-A_negative_)] × 100; <5% is generally considered acceptable.

### 2.14 Statistical analysis

Statistical analysis was performed using one-way analysis of variance (ANOVA) followed by Tukey’s *post hoc* test for multiple comparisons. Data are presented as mean ± SD from three independent experiments. *p* < 0.05 (*), *p* < 0.01 (**) and *p* < 0.001 (***) were used to indicate statistical difference.

## 3 Results

### 3.1 Preparation and characterization of NPs

**FIGURE 1:**
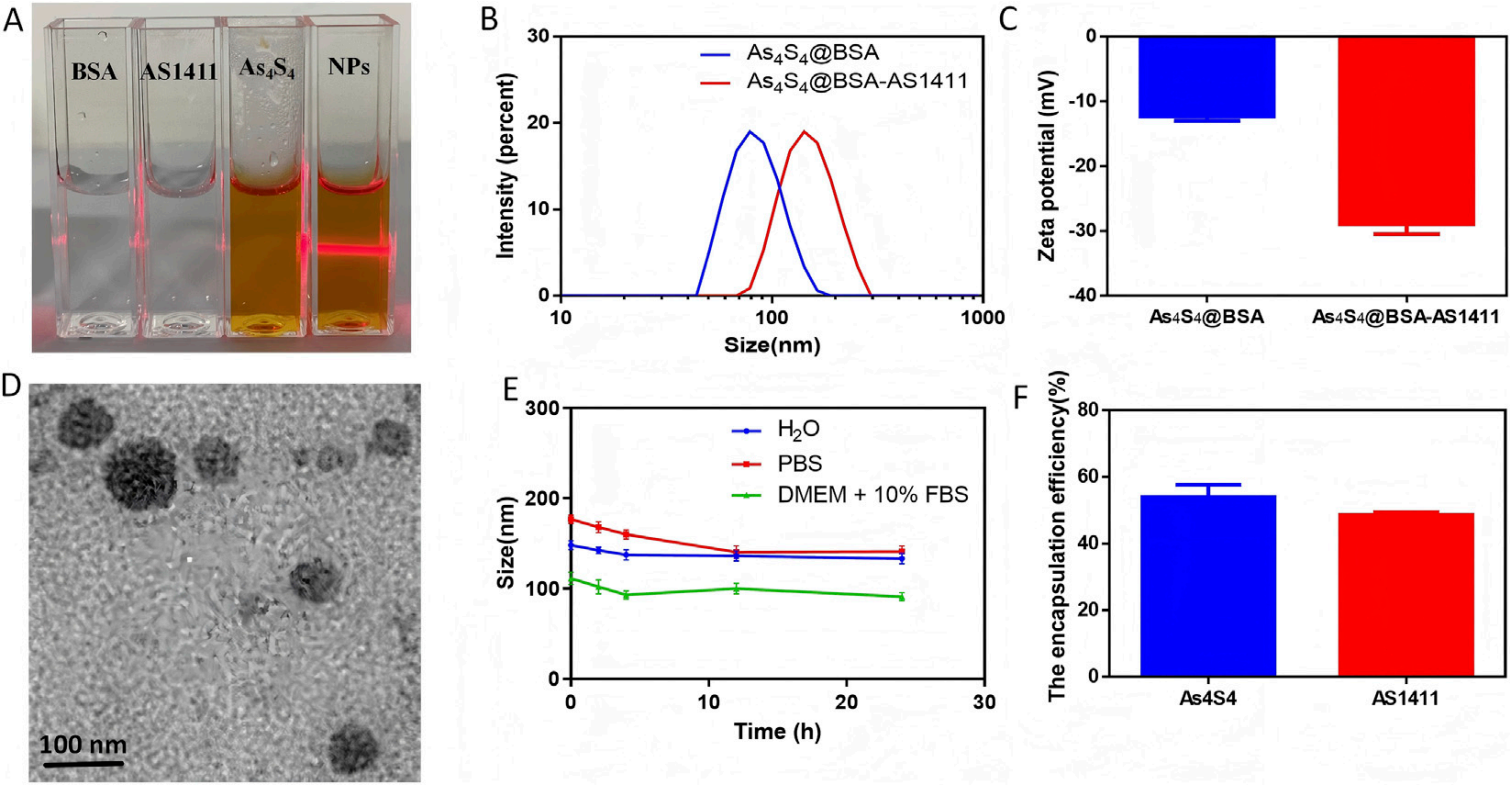
**(A)** Tyndall effect of PBS, free AS1411, free As_4_S_4_, and As_4_S_4_@BSA-AS1411 NPs under laser irradiation. **(B)** Particle sizes of As_4_S_4_@BSA NPs and As_4_S_4_@BSA-AS1411 NPs. **(C)** ζ-potentials of As_4_S_4_@BSA NPs and As_4_S_4_@BSA-AS1411 NPs. **(D)** TEM image of As_4_S_4_@BSA-AS1411 NPs. **(E)** Stability ofAs_4_S_4_@BSA-AS1411 NPs in H_2_O, DMEM cell medium (with 10% FBS), and PBS at 37 °C over a period of 24 h incubation. **(F)** Encapsulation efficiency of As_4_S_4_ and conjugation efficiency of AS1411 in the As_4_S_4_@BSA-AS1411 NPs.

As_4_S_4_@BSA-AS1411 NPs were prepared via a bottom-up assembly using BSA as the stabilizing matrix and AS1411 as the targeting ligand at a BSA: As_4_S_4_ weight ratio of 3 : 1 (Wang et al., 2021). The nanoparticle dispersion exhibited a clear Tyndall effect (Figure 1A). DLS analysis showed that the hydrodynamic diameter increased from 76.2 ± 8.15 nm for As_4_S_4_@BSA to 135.5 ± 7.79 nm for As_4_S_4_@BSA-AS1411, with ζ-potentials shifting from −11.13 ± 1.78 mV to −29.07 ± 2.68 mV (Figures 1B,C; Supplementary Figure S1). TEM revealed a near-spherical morphology, with a dry-state diameter consistent with the DLS measurements (Figure 1D). Batch-to-batch reproducibility was assessed using three independently prepared batches, particle size and PDI showed acceptable consistency (Supplementary Figures S1, S2). Additionally, no obvious changes in particle size were observed for these three batches upon storage in water, PBS, and complete cell culture medium over 24 h (Figure 1E). Quantification of conjugation efficiencies showed that 53.4% ± 2.1% of the initial As_4_S_4_ was encapsulated within the nanoparticle core, and the conjugation efficiency of AS1411 was 47.8% ± 3.5% (Figure 1F). The anchorage stability of the AS1411 aptamer on the NP surface was further evaluated. Supernatants were collected at 0, 6, 12, 24, 48, and 72 h post-incubation in 50% serum and run on a gel (Supplementary Figure S3). No free AS1411 band was seen up to 48 h, while a weak band appeared at 72 h.

### 3.2 *In vitro* tumor-targeted delivery

**FIGURE 2:**
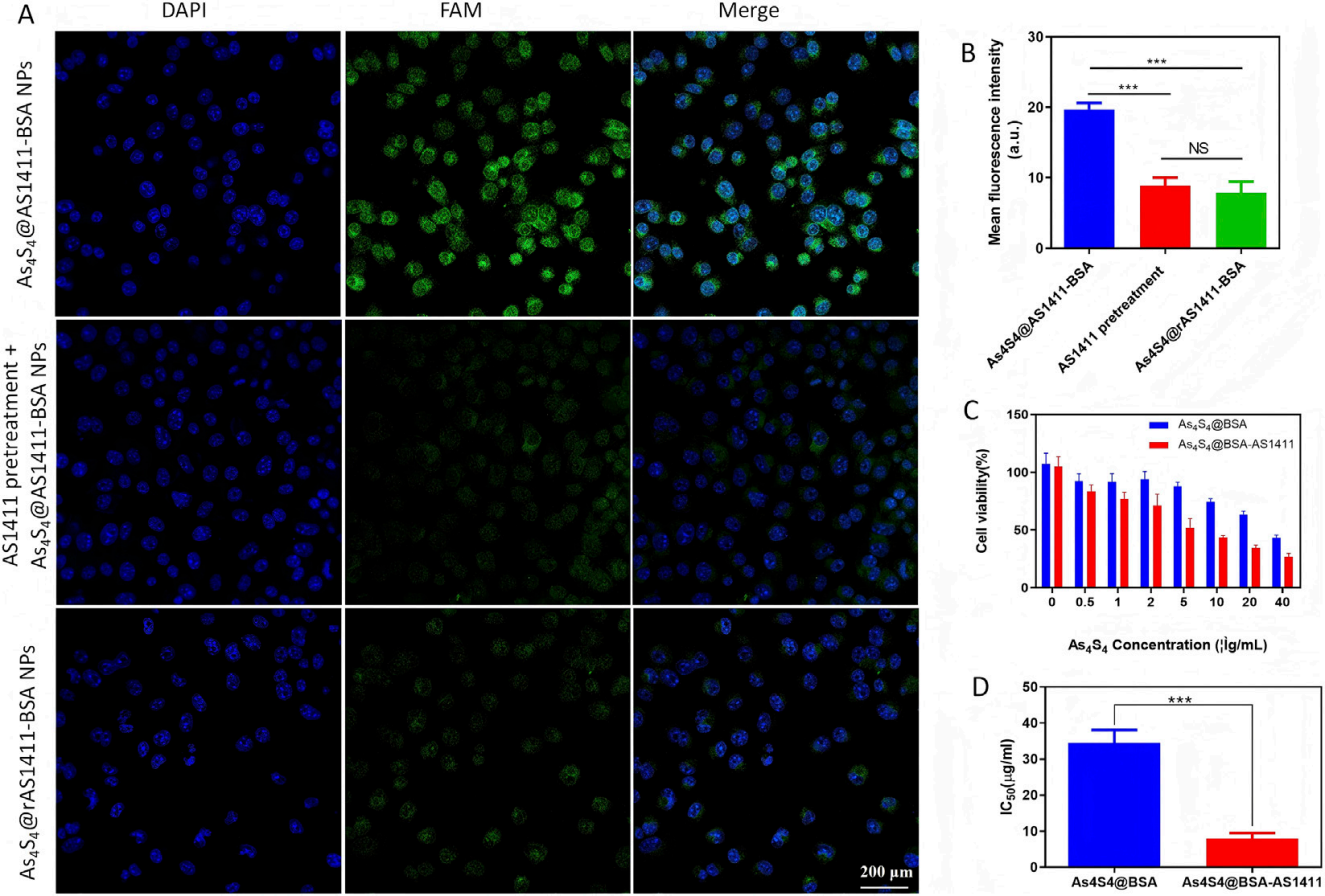
**(A)** Fluorescence imaging of 4T1 cells after incubation with As_4_S_4_@BSA-AS1411 and As_4_S_4_@BSA-rAS1411 NPs, with or without free AS1411 pre-incubation. **(B)** Quantitative analysis of cellular uptake fluorescence intensity in 4T1 cells. Fluorescence was normalized to the group without competitor. **(C)** Concentration-dependent cytotoxicity of As_4_S_4_@BSA and As_4_S_4_@BSA-AS1411 NPs in 4T1 cells determined by MTT assay. Cells were treated with various concentrations (0–40 μg/mL) for 24 h **(D)** IC_50_ values of As_4_S_4_@BSA NPs and As_4_S_4_@BSA-AS1411 NPs in 4T1 cells.

FAM fluorescence was used as a tracer for cellular imaging. 4T1 cells treated with As_4_S_4_@BSA-AS1411 showed stronger intracellular green fluorescence than cells treated with As_4_S_4_@BSA-rAS1411. Pre-incubation with free AS1411 (50 μmol/L, 30 min) reduced the intracellular fluorescence of As_4_S_4_@BSA-AS1411 to a level comparable to that of As_4_S_4_@BSA-rAS1411 (Figure 2A). Quantification of the fluorescence intensity (Figure 2B) gave a 58% decrease for the free AS1411 pre-incubation group relative to the group without competitor.

### 3.3 *In vitro* antitumor efficacy

**FIGURE 3:**
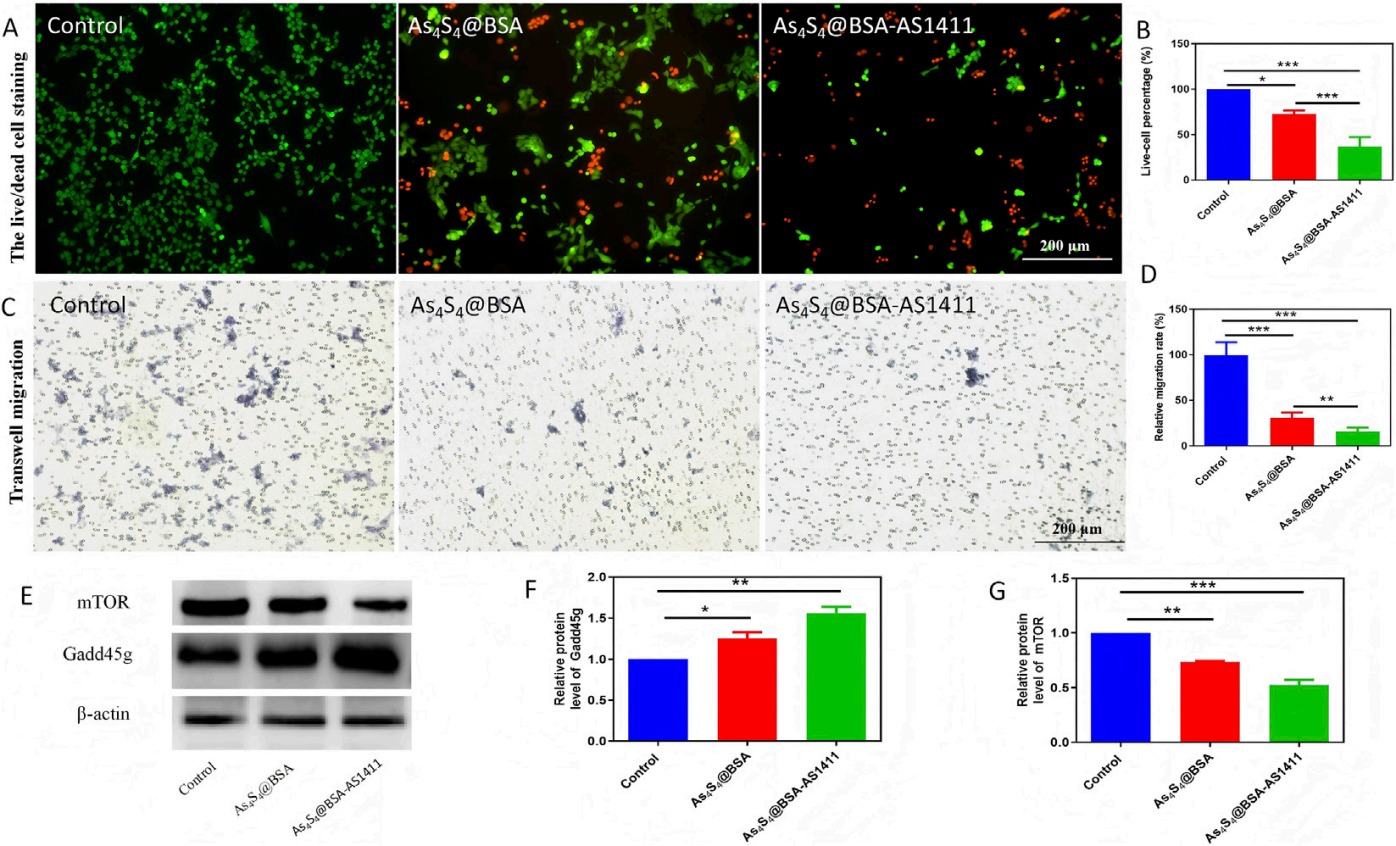
Effects of As_4_S_4_@BSA-AS1411 NPs on migration and protein expression in 4T1 cells. **(A,B)** Live/dead staining and quantitative analysis; **(C,D)** Transwell migration assay and quantitative analysis; **(E)** Western blot analysis of GADD45G and mTOR; **(F,G)** densitometric quantification of GADD45G and mTOR expression.

4T1 cell viability was assessed by MTT assay. Both As_4_S_4_@BSA and As_4_S_4_@BSA-AS1411 NPs reduced cell viability in a concentration-dependent manner (Figure 2C). The IC_50_ value for As_4_S_4_@BSA-AS1411 was 7.93 ± 1.53 μg/mL, compared with 34.44 ± 3.67 μg/mL for As_4_S_4_@BSA (Figure 2D). Calcein-AM/PI staining showed that both NPs formulations increased the number of PI-positive cells and decreased Calcein-AM-positive cells relative to the PBS control. Quantification of the live-cell to total-cell ratio showed that As_4_S_4_@BSA-AS1411-treated cultures had a lower live-cell percentage than As_4_S_4_@BSA-treated cultures (Figures 3A,B).

Transwell migration assays showed that As_4_S_4_@BSA-AS1411 NPs more effectively reduced 4T1 cell migration than non-targeted As_4_S_4_@BSA NPs (Figures 3C,D), indicating enhanced suppression of migratory potential. To determine whether this reduction was associated with treatment-induced cytotoxicity, cell viability was evaluated under the same concentration and exposure duration used in the migration assay. The MTT results showed that cell viability remained above 85% in all treatment groups after 12 h (Supplementary Figure S4), indicating that the decreased number of migrated cells was not primarily attributable to reduced cell survival. Therefore, the stronger anti-migratory effect of As_4_S_4_@BSA-AS1411 NPs likely reflected genuine suppression of migratory ability rather than nonspecific cytotoxicity.

Western blot analysis was performed to examine the expression of GADD45G and mTOR in 4T1 cells after different treatments. Compared with the PBS group, As_4_S_4_@BSA NPs increased GADD45G expression and reduced mTOR protein levels to a certain extent, whereas As_4_S_4_@BSA-AS1411 NPs produced a more pronounced effect (Figure 3E). Quantitative analysis showed that GADD45G expression was significantly upregulated in the As_4_S_4_@BSA-AS1411 NPs group, while mTOR expression was markedly downregulated compared with the non-targeted As_4_S_4_@BSA NPs group (Figures 3F,G). These results indicate that AS1411 functionalization enhanced the regulatory effect of As_4_S_4_-loaded nanoparticles on GADD45G and mTOR expression in 4T1 cells.

### 3.4 *In vivo* distribution and tumor targeting

**FIGURE 4:**
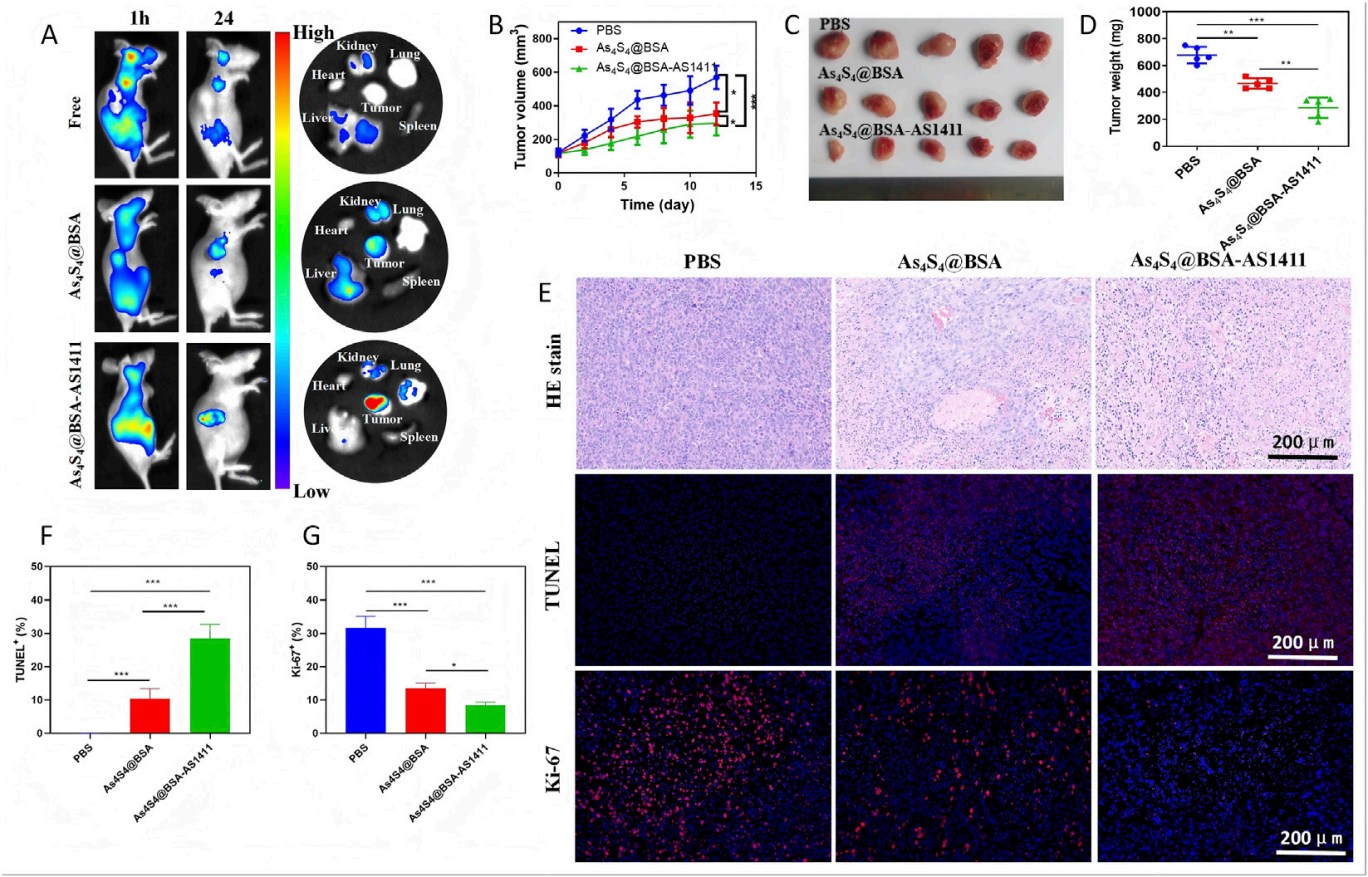
*In vivo* distribution and antitumor activity of As_4_S_4_@BSA-AS1411 NPs in 4T1 tumor-bearing mice. **(A)** *In vivo* fluorescence images at 1 h and 24 h after intravenous injection, and *ex vivo* images of tumors and major organs (heart, liver, spleen, lung, kidney) at 24 h. **(B)** Tumor growth curves during 14-day treatment (n = 5). **(C)** Representative tumor images at the endpoint. **(D)** Tumor weights at the endpoint (n = 5). **(E)** Representative images of H&E staining, TUNEL immunofluorescence, and Ki67 immunofluorescence in tumor sections. **(F)** Quantification of TUNEL-positive cells per field (n = 3). **(G)** Quantification of Ki67-positive cells per field (n = 3).

*In vivo* imaging in 4T1 tumor-bearing nude mice showed systemic fluorescence at 1 h after injection across all groups (Figure 4A). At 24 h, As_4_S_4_@BSA-AS1411 NPs exhibited stronger tumor-associated fluorescence than scrambled rAS1411 NPs (Figure 4A). *Ex vivo* imaging at 24 h further confirmed higher tumor accumulation of As_4_S_4_@BSA-AS1411 NPs, whereas rAS1411 NPs showed more pronounced signals in the liver and kidney (Figure 4A). Quantitative analysis of the excised tumor tissues showed that the fluorescence intensity of tumors in the As_4_S_4_@BSA-AS1411 NPs group was 4.4-fold higher than that in the As_4_S_4_@BSA-rAS1411 NPs group, with a statistically significant difference (Supplementary Figure S5). These results demonstrate that AS1411 functionalization enhanced the tumor accumulation of nanoparticles.

### 3.5 *In vivo* antitumor efficacy

To evaluate *in vivo* antitumor efficacy, 4T1 tumor–bearing BALB/c nude mice were randomized into three groups: PBS, As_4_S_4_@BSA NPs, and As_4_S_4_@BSA-AS1411 NPs. Treatments were administered intravenously at an As_4_S_4_ dose of 10 mg/kg at 3-day intervals (days 0, 3, 6, 9, and 12). Both nanoparticle formulations inhibited tumor growth versus PBS, with As_4_S_4_@BSA-AS1411 NPs showing the greatest suppression (Figure 4B). Consistently, endpoint tumor photographs showed smaller tumor sizes in the nanoparticle-treated groups, particularly in the As_4_S_4_@BSA-AS1411 NPs group (Figure 4C). Tumor weight analysis further confirmed this trend, showing a significant reduction in tumor weight after As_4_S_4_@BSA-AS1411 NPs treatment compared with PBS and non-targeted As_4_S_4_@BSA NPs (Figure 4D).

Histological analysis of tumor tissues further supported the enhanced antitumor effect of the targeted nanoparticles. H&E staining showed dense tumor cell arrangement and obvious nuclear morphology in the PBS group, whereas nanoparticle treatment induced varying degrees of tumor tissue damage. The As_4_S_4_@BSA-AS1411 NPs group exhibited more extensive histopathological changes, including necrosis and nuclear fragmentation (Figure 4E).

TUNEL and Ki67 immunofluorescence staining were further performed to evaluate apoptosis and proliferation in tumor tissues. TUNEL staining showed only weak apoptotic signals in the PBS group, while stronger red fluorescence was observed in the nanoparticle-treated groups, with the most intense TUNEL-positive signal detected in the As_4_S_4_@BSA-AS1411 NPs group (Figure 4E). Quantitative analysis showed that the percentage of TUNEL-positive cells was significantly increased in the As_4_S_4_@BSA-AS1411 NPs group compared with the PBS and As_4_S_4_@BSA NPs groups (Figure 4F). In contrast, Ki67 staining revealed abundant proliferating cells in the PBS group, whereas Ki67-positive signals were reduced after nanoparticle treatment and were lowest in the As_4_S_4_@BSA-AS1411 NPs group (Figure 4E). Quantification of Ki67-positive cells further confirmed that As_4_S_4_@BSA-AS1411 NPs significantly suppressed tumor cell proliferation compared with the other groups (Figure 4G). These results demonstrate that As_4_S_4_@BSA-AS1411 NPs exerted the strongest *in vivo* antitumor effect, accompanied by increased tumor cell apoptosis and reduced proliferative activity.

### 3.6 Safety and biocompatibility

**FIGURE 5:**
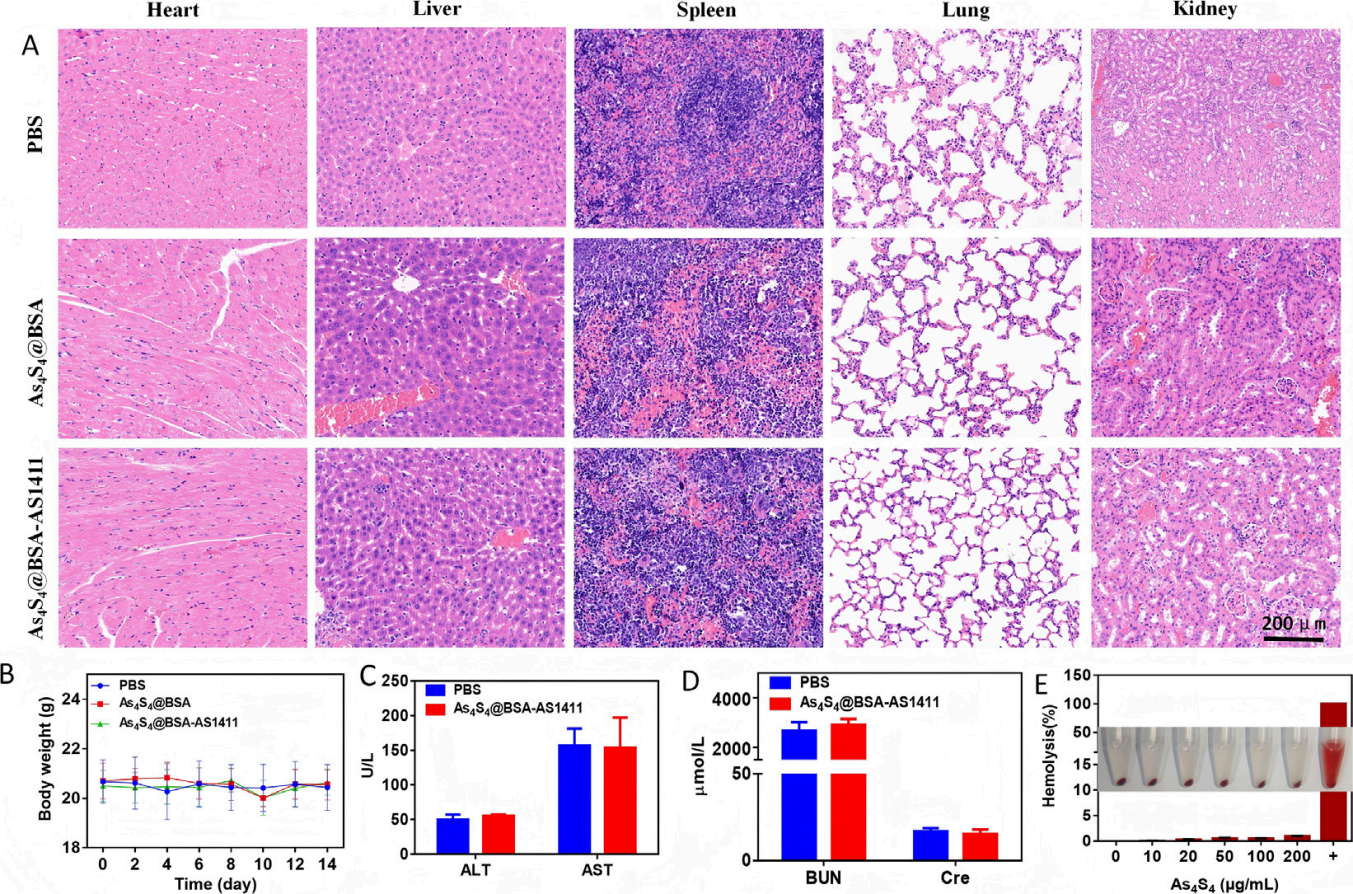
**(A)** Histological analysis of the heart, liver, spleen, lung and kidney of mice after 14 days treatment with different drugs. **(B)** Bodyweight change of mice during each treatment. **(C)** Serum ALT and AST levels in mice from the PBS and As_4_S_4_@BSA-AS1411 NPs. **(D)** Serum BUN and Cre levels in mice from the PBS and As_4_S_4_@BSA-AS1411 NPs. **(E)** Hemolysis rates of As_4_S_4_@BSA-AS1411 NPs at different concentrations, with PBS and 0.1% Triton X-100 as the negative and positive controls, respectively.

To evaluate the *in vivo* safety of the nanoparticles, body weight, major organ histology, serum biochemical parameters, routine hematological indices, and hemolytic activity were examined. During the 14-day treatment period, no significant body weight loss was observed in mice treated with As_4_S_4_@BSA NPs or As_4_S_4_@BSA-AS1411 NPs compared with the PBS group (Figure 5B). H&E staining of major organs, including the heart, liver, spleen, lungs, and kidneys, showed normal tissue architecture in all groups, with no obvious inflammatory infiltration, degeneration, necrosis, or other pathological abnormalities (Figure 5A).

Serum biochemical analysis showed that the levels of liver function markers, including ALT and AST, and renal function markers, including BUN and Cre, were not significantly different between the As_4_S_4_@BSA-AS1411 NPs group and the PBS group (Figures 5C,D). Routine hematological analysis further showed no significant differences in the measured hematological parameters among the PBS, As_4_S_4_@BSA NPs, and As_4_S_4_@BSA-AS1411 NPs groups(Supplementary Figure S6). These results indicate that repeated intravenous administration of the NPs did not cause obvious hepatic, renal, or hematological toxicity under the tested conditions.

In addition, the hemolysis assay showed that As_4_S_4_@BSA-AS1411 NPs induced less than 5% hemolysis at all tested concentrations (Figure 5E), indicating good blood compatibility *in vitro*. Collectively, these results demonstrate that As_4_S_4_@BSA-AS1411 NPs exhibited a favorable preliminary safety profile *in vivo* and did not induce obvious systemic toxicity during the treatment period.

## 4 Discussion

The clinical translation of arsenic therapeutics faces a long-standing physicochemical hurdle. Tetra-arsenic tetrasulfide (As_4_S_4_) is highly hydrophobic, tends to aggregate in aqueous media, and may undergo rapid opsonization and reticuloendothelial clearance after systemic administration. This paradox has historically narrowed the therapeutic window of arsenic agents, restricting their use largely to selected hematological malignancies (Wang et al., 2008; Wu et al., 2011; Zhu et al., 2018). Serum albumin, as a biocompatible protein carrier with hydrophobic-binding domains, offers a natural strategy to improve the pharmaceutical behavior of hydrophobic cargos (Rabbani and Ahn, 2019). In our system, BSA served as a stabilizing matrix for As_4_S_4_ clusters, improving their aqueous dispersibility and colloidal behavior under biologically relevant conditions. The observed colloidal and serum stability is important for maintaining nanoparticle dispersion during biological exposure and preserving the availability of surface-conjugated AS1411 for target recognition. Unlike many synthetic polymeric carriers that require complex formulation procedures, this albumin-based approach exploits the biocompatibility and transport-related properties of a protein carrier. This concept is supported by the clinical success of albumin-based formulations such as Abraxane®, although the present BSA-based arsenic formulation will require further optimization before translational development (Elzoghby et al., 2012; Solanki et al., 2021). It should be noted that, in the present formulation, As_4_S_4_ loading mainly relies on albumin-assisted stabilization rather than direct covalent conjugation between the drug and carrier, whereas AS1411 is subsequently attached to the nanoparticle surface through EDC/NHS-mediated coupling. This separation of drug encapsulation and targeting-ligand conjugation provides a relatively straightforward strategy for constructing an albumin-based arsenic nanomedicine. The versatility of albumin as a carrier for arsenic species has been increasingly recognized. Yang et al. recently reported a dual-drug-conjugated human serum albumin nanoparticle system co-delivering arsenic trioxide and all-trans retinoic acid, which achieved dual-site spatiotemporal inhibition of the oncogenic regulator Pin1 and produced synergistic antitumor effects against hepatocellular carcinoma with favorable safety profiles (Yang et al., 2025). In parallel, Wang et al. demonstrated that folic acid-modified albumin-stabilized arsenic trioxide nanoparticles, when co-encapsulated with Beclin 1-targeting DNAzyme, shifted the cell death modality from apoptosis to pyroptosis via the caspase-3-GSDME pathway, significantly enhancing therapeutic outcomes in liver cancer models (Wang et al., 2025). These advances underscore the potential of albumin as a versatile platform for arsenic-based nanomedicines. Recent advances in arsenic-based nanomedicines, including various formulation strategies for arsenic species, have also been reported (Chen et al., 2006; Bujňáková et al., 2015). In this context, our As_4_S_4_@BSA-AS1411 system represents a distinct approach that combines albumin-based stabilization with AS1411-mediated active targeting for triple-negative breast cancer.

Passive tumor accumulation alone, however, is unlikely to fully account for the enhanced therapeutic activity observed with this payload. The non-targeted As_4_S_4_@BSA formulation showed a much higher IC_50_ than the targeted version, whereas AS1411 functionalization decreased the IC_50_ from 34.44 ± 3.67 μg/mL to 7.93 ± 1.53 μg/mL. This difference suggests that the enhanced activity of As_4_S_4_@BSA-AS1411 NPs is not simply a consequence of nanoparticle formulation, but is closely related to active cellular recognition and internalization. Nucleolin is aberrantly overexpressed on the surface of 4T1 cells and many other malignant cells, and AS1411-conjugated NPs are generally considered to undergo nucleolin-associated cellular uptake, which may differ from the nonspecific endocytic pathways involved in untargeted particle internalization (Soundararajan et al., 2009; Reyes-Reyes et al., 2010; Fonseca et al., 2015; Van Den Avont and Sharma-Walia, 2023). In our study, scrambled rAS1411-modified nanoparticles showed weaker cellular uptake, while pre-incubation with excess free AS1411 reduced the fluorescence intensity of As_4_S_4_@BSA-AS1411 NPs by approximately 58%. These findings support the involvement of AS1411–nucleolin recognition in the enhanced internalization of the targeted nanoparticles. This mechanistic distinction suggests that the intracellular delivery route of the payload, rather than nanoparticle exposure alone, may influence whether downstream stress-response pathways are efficiently engaged (Chen et al., 2023). We acknowledge, however, that targeted and non-targeted nanoparticles may also differ in intracellular release kinetics, possibly due to distinct endosomal pH environments or enzymatic degradation rates. This interpretation is consistent with previous observations that non-targeted nanoparticles can achieve appreciable cellular uptake yet fail to mediate effective cytoplasmic cargo release due to entrapment within late endosomes or lysosomes (Cheng et al., 2025). Distinguishing between these possibilities will inform whether the enhanced potency of the targeted formulation arises primarily from receptor-associated uptake or from more favorable intracellular release kinetics.

This observation raises a mechanistic question worth closer attention. Non-targeted nanoparticles, despite delivering some arsenic into cells through nonspecific endocytosis, induced weaker GADD45G upregulation and less reduction of total mTOR protein expression than the targeted formulation. This finding does not fit a simple “more drug, more effect” model; rather, it suggests that the intracellular delivery route may influence pathway regulation. We hypothesize that As_4_S_4_ clusters internalized through nonspecific endocytic pathways may become partly trapped in endolysosomal compartments, where acidic pH and hydrolytic enzymes may either degrade the BSA carrier or sequester arsenic species away from their cytosolic and nuclear targets (Sun and Jiang, 2023). In contrast, AS1411-mediated nucleolin engagement may facilitate more efficient intracellular delivery, allowing released arsenic species to more effectively induce GADD45G-associated stress responses and reduce mTOR-related growth signaling (Saxton and Sabatini, 2017). GADD45G has been established as a tumor suppressor that initiates cellular stress responses and inhibits breast cancer cell carcinogenesis, while mTOR signaling serves as a central hub integrating growth and metabolic cues (Saxton and Sabatini, 2017; Zhang et al., 2021). Mechanistically, arsenic species have been shown to induce GADD45 family members through nucleolin-dependent mRNA stabilization and an internal ribosome entry site (IRES)-dependent translational mechanism, bypassing the cap-dependent translation machinery (Chang et al., 2007). This finding raises the possibility that the enhanced GADD45G upregulation observed with our targeted nanoparticles may result from both increased intracellular arsenic delivery and the intrinsic ability of arsenic to interact with nucleolin-related regulatory processes. This hypothesis requires formal validation through endocytic inhibitor studies and subcellular arsenic quantification by ICP-MS. The feasibility of such an approach has been established in previous studies that employed size-exclusion chromatography coupled with ICP-MS to profile arsenic distribution in cytosol fractions and differential centrifugation protocols combined with HPLC-ICP-MS to quantify arsenic compartmentalization across subcellular organelles (Pizarro et al., 2004). Future studies should include quantitative pH-dependent release assays and subcellular arsenic speciation analysis by ICP-MS to distinguish between these possibilities. If confirmed, this insight would carry implications for nanomedicine design: for drugs acting on intracellular non-nuclear targets, optimizing subcellular addressability may be every bit as critical as achieving tumor-level accumulation.

Rather than relying on indirect numerical comparisons with free arsenic trioxide or liposomal arsenic formulations, which can be influenced by differences in cell type, assay duration, drug valence state, and formulation composition, the present data more directly demonstrate the advantage of AS1411 functionalization within the same experimental system. Compared with non-targeted As_4_S_4_@BSA NPs, As_4_S_4_@BSA-AS1411 NPs showed stronger cytotoxicity, greater inhibition of cell migration under conditions with cell viability above 85%, enhanced tumor accumulation, and superior tumor growth suppression. In breast cancer, recent redox-responsive nanoassembly strategies have likewise demonstrated that rational formulation design can influence nanoparticle stability, drug release, pharmacokinetics, antitumor efficacy, and systemic toxicity (Yuan et al., 2025). Notably, a previous study using arsenite-loaded albumin nanoparticles for hepatocellular carcinoma demonstrated the feasibility of albumin as a carrier for arsenic species, although that work relied on a different arsenic form and a different tumor model (Zhang et al., 2022). In the present study, the enhanced antitumor activity of the targeted formulation was achieved without obvious short-term systemic toxicity. Serum alanine aminotransferase (ALT) and aspartate aminotransferase (AST), which are standard biochemical markers for hepatocyte injury, and blood urea nitrogen (BUN) and creatinine (Cre), which are routinely used to monitor renal function, showed no significant differences from PBS controls under the tested dosing regimen (Gu et al., 2025). These findings suggest that AS1411-guided albumin delivery may improve the therapeutic performance of As_4_S_4_ while maintaining a favorable preliminary safety profile.

The *in vivo* data provide cross-scale support for this targeting and therapeutic framework. In 4T1 tumor-bearing mice, As_4_S_4_@BSA-AS1411 NPs displayed stronger tumor-associated fluorescence at 24 h after injection than scrambled rAS1411-conjugated controls. *Ex vivo* fluorescence analysis further showed that the fluorescence intensity of excised tumors in the As_4_S_4_@BSA-AS1411 group was 4.4-fold higher than that in the rAS1411 group. This result supports the contribution of AS1411-mediated active targeting to improved tumor accumulation. In terms of antitumor efficacy, the targeted formulation suppressed tumor growth, reduced endpoint tumor weights, induced more extensive tumor tissue damage on H&E staining, produced stronger TUNEL-positive apoptotic signals, and markedly decreased Ki67-positive proliferative signals. Quantitative analysis of TUNEL and Ki67 staining further confirmed increased apoptosis and reduced proliferative activity in the As_4_S_4_@BSA-AS1411 group. These *in vivo* findings are consistent with the enhanced cellular uptake, cytotoxicity, and GADD45G/mTOR-related changes observed *in vitro*, suggesting that improved tumor accumulation and tumor-cell internalization contributed to the stronger therapeutic response. The non-targeted formulation showed weaker activity, reinforcing that albumin-based stabilization alone, although beneficial for formulation properties, is insufficient to maximize therapeutic efficacy without an additional active targeting component.

Given arsenic’s well-established toxicity profile, the safety data from this 14-day study are noteworthy. Treated mice exhibited no significant body weight loss, no histopathological abnormalities in major organs, and serum biochemical parameters comparable to those of PBS-treated mice, collectively indicating that the targeted nanoformulation did not cause evident short-term systemic toxicity. Routine hematological analysis further showed no significant differences among the PBS, As_4_S_4_@BSA NPs, and As_4_S_4_@BSA-AS1411 NPs groups, suggesting that repeated intravenous administration did not induce obvious hematological toxicity under the tested conditions. The hemolysis assay showed <5% lysis across all tested concentrations, confirming favorable blood compatibility (Nalezinková, 2020). This favorable safety profile may be related to the BSA-based carrier, which can reduce nonspecific interactions with blood components, and to AS1411-mediated targeting, which may help limit nonspecific exposure of normal tissues. This combination provides a basis for improving the therapeutic index of As_4_S_4_, a drug class historically limited by narrow safety margins (Flora, 2011; Zhu et al., 2018). Nevertheless, longer-term safety evaluation is still required to determine whether this favorable profile can be maintained after extended or repeated dosing.

Before turning to the specific limitations of this study, we wish to briefly highlight several translational issues that warrant consideration. First, the metabolic fate of As_4_S_4_ *in vivo* is largely unknown in our current setting—specifically, its conversion to methylated species such as MMA and DMA, and how these metabolites distribute across and clear from different tissues. Systematic speciation analysis using HPLC-ICP-MS in future pharmacokinetic studies will be needed to address this gap. Second, although albumin is a biocompatible protein carrier, the use of BSA and the conjugation of AS1411 could in principle trigger immune responses after repeated dosing; monitoring anti-BSA and anti-AS1411 antibody titers in long-term studies would help clarify this risk. Third, although our laboratory-scale batches showed acceptable physicochemical consistency, further formulation optimization is still needed before translational development. In particular, the reproducibility of particle size, AS1411 conjugation efficiency, drug loading, and serum stability should be evaluated in larger-scale preparations. In addition, lyophilized storage conditions and reconstitution stability need to be systematically investigated to determine whether the formulation can maintain its colloidal properties and targeting functionality during long-term storage.

Several limitations of this study should be acknowledged and define clear priorities for future work. Mechanistically, we have not established a causal hierarchy between GADD45G upregulation and total mTOR downregulation; rescue experiments using mTOR pathway modulation or siRNA-mediated GADD45G silencing will be necessary to determine whether these pathways operate in parallel or in a linear cascade. Given that arsenic-based nanotherapeutics may engage more than one regulated cell-death program, future studies should distinguish apoptosis from pyroptosis using complementary markers, including cleaved caspase-3, cleaved PARP, and GSDME-related assays (Li et al., 2022). Similarly, the proposed endocytic pathway specificity requires formal confirmation with pharmacological inhibitors such as amiloride, chlorpromazine, or methyl-β-cyclodextrin, combined with subcellular arsenic quantification by ICP-MS. The alternative interpretation of differential release kinetics, as noted above, also demands experimental resolution. From a formulation standpoint, quantitative pH-dependent release profiles and long-term stability data under lyophilized storage remain essential for translational planning. It should also be noted that the discrepancy between hydrodynamic diameter measured by DLS and the dry-state diameter observed by TEM is commonly observed in nanoparticle characterization, reflecting the different physical principles of these two techniques, and does not compromise the validity of the colloidal stability assessment (Filippov et al., 2023). Regarding the biological model, the present *in vivo* study was performed in a 4T1 tumor-bearing nude mouse model, which does not fully reproduce the immune microenvironment of immunocompetent or orthotopic TNBC models. Validation in orthotopic breast cancer models, immunocompetent models, or patient-derived xenografts will be critical to assess tumor heterogeneity and stromal influences on targeting efficiency (Bianchini et al., 2016; Zagami and Carey, 2022). Finally, extended toxicology studies beyond 14 days and pharmacokinetic assessment in more clinically relevant models will be needed in later-stage translational studies. Although the current study incorporated routine hematological analysis, longer-term observation, such as 28 days or beyond, will be essential to fully assess potential cumulative toxicity and long-term safety (Du et al., 2018; Zhu et al., 2022). Recent developments in arsenic-based nanotherapeutics, including arsenic trioxide nanoparticles for pyroptosis activation, indicate that this field is rapidly evolving, and our As_4_S_4_@BSA-AS1411 platform adds a distinct nucleolin-targeted strategy to the expanding arsenal of arsenic nanomedicines (Wang et al., 2025). Addressing these points will not only refine the mechanistic model but also establish a more robust foundation for translation. More broadly, our findings highlight subcellular addressability as a design parameter that may be as consequential as tumor-level targeting for drugs that act on intracellular sensors.

## 5 Conclusion

In summary, we developed a nucleolin-targeted realgar nanomedicine by encapsulating As_4_S_4_ clusters within bovine serum albumin and functionalizing the surface with the AS1411 aptamer (As_4_S_4_@BSA-AS1411 NPs). The nanoparticles showed good colloidal stability and enhanced tumor-selective cellular uptake. Compared with non-targeted controls, AS1411 modification markedly increased cytotoxicity and inhibited 4T1 cell migration, accompanied by GADD45G upregulation and mTOR suppression. *In vivo* imaging confirmed preferential tumor accumulation, and systemic dosing produced pronounced tumor growth inhibition with increased apoptosis and reduced proliferation. Importantly, the formulation exhibited minimal systemic toxicity and negligible hemolysis, supporting its translational potential.

## Data Availability

The raw data supporting the conclusions of this article will be made available by the authors, without undue reservation.
