# Supplementary material for: AS1411-functionalized albumin nanoparticles for nucleolin-targeted realgar delivery and therapy in triple-negative breast cancer
Source: Front Pharmacol. 2026 Jul 29;17:1891302. doi: 10.3389/fphar.2026.1891302 (PMC13462193; doi:10.3389/fphar.2026.1891302)
Supplement: Supplementary file 1 [file Supplementaryfile1.docx]

Supplementary Material

**Figure S1.** Hydrodynamic diameters of As₄S₄@BSA NPs and As₄S₄@BSA-AS1411 NPs. Data are presented as mean ± SD, n = 3.

**Figure S2.** Polydispersity index (PDI) of As₄S₄@BSA NPs and As₄S₄@BSA-AS1411 NPs. Data are presented as mean ± SD, n = 3.

**Figure S3.** Serum stability evaluation of As₄S₄@BSA-AS1411 NPs.

**Figure S4.** MTT assay for cell viability at 12 h under the concentrations applied in Transwell migration experiments.

**Figure S5.** Quantitative analysis of ex vivo tumor fluorescence intensity at 24 h post-intravenous administration of nanoparticles.

**Figure S6.** Complete blood count (CBC) analysis.
